# Ameliorating effects of aged garlic extracts against Aβ-induced neurotoxicity and cognitive impairment

**DOI:** 10.1186/1472-6882-13-268

**Published:** 2013-10-18

**Authors:** Ji Hee Jeong, Hee Rok Jeong, Yu Na Jo, Hyeon Ju Kim, Jeong Hae Shin, Ho Jin Heo

**Affiliations:** 1 Division of Applied Life Science, Institute of Agriculture and Life Science, Gyeongsang National University, Jinju 660-701, Korea; 2 Department of Analytical research, Pacificpharma Corporation, Anseong 456-370, Republic of Korea; 3 Namhae Garlic Research Institute, Namhae South Korea

**Keywords:** Aged garlic, Amyloid beta protein, Cognitive impairment, Dimethyl disulfide, Neurotoxicity

## Abstract

**Background:**

*In vitro* antioxidant activities and neuron-like PC12 cell protective effects of solvent fractions from aged garlic extracts were investigated to evaluate their anti-amnesic functions. Ethyl acetate fractions of aged garlic had higher total phenolics than other fractions.

**Methods:**

Antioxidant activities of ethyl acetate fractions from aged garlic were examined using 2,2'-azino-bis(3-ethylbenzthiazoline-6-sulfonic acid) diammonium salt (ABTS) and malondialdehyde (MDA) inhibitory effect using mouse whole brain homogenates. Levels of cellular oxidative stress as reactive oxygen species (ROS) accumulation were measured using 2',7'-dichlorofluorescein diacetate (DCF-DA). PC12 cell viability was investigated by 3-[4,5-dimethythiazol-2-yl]-2,5-diphenyl tetrazolium bromide (MTT) and lactate dehydtrogenase (LDH) assay. The learning and memory impairment in institute of cancer research (ICR) mice was induced by neurotoxic amyloid beta protein (Aβ) to investigate *in vivo* anti-amnesic effects of aged garlic extracts by using Y-maze and passive avoidance tests.

**Results:**

We discovered that ethyl acetate fractions showed the highest ABTS radical scavenging activity and MDA inhibitory effect. Intracellular ROS accumulation resulting from Aβ treatment in PC12 cells was significantly reduced when ethyl acetate fractions were presented in the medium compare to PC12 cells which was only treated with Aβ only. Ethyl acetate fractions from aged garlic extracts showed protection against Aβ-induced neurotoxicity. Pre-administration with aged garlic extracts attenuated Aβ-induced learning and memory deficits in both *in vivo* tests.

**Conclusions:**

Our findings suggest that aged garlic extracts with antioxidant activities may improve cognitive impairment against Aβ-induced neuronal deficit, and possess a wide range of beneficial activities for neurodegenerative disorders, notably Alzheimer's disease (AD).

## Background

According to the World Health Organization (WHO), 5% of men and 6% of women aged above 60 years suffer from dementia such as Alzheimer's disease (AD). In addition, AD can also afflict young individual as early as 40 years of age [1]. AD is one of the most serious threats to human health in aged societies of developed countries, and is one of the major neurodegenerative diseases, which is characterized as loss of memory and cognition. In particular, AD is a progressive disease specified in the brain by the presence of senile plaques rich in insoluble aggregates of amyloid beta protein (Aβ) and neurofibrillary tangles [2]. Accumulated intracellular Aβ-induced oxidative stress including H_2_O_2_ causes peroxidation of membrane lipids and apoptotic cell death by activation of caspases [3,4]. These reactive oxygen species (ROS) also make inflammation or lesions on various organs, and are associated with various degenerative diseases including cancer, aging, arteriosclerosis and neurodegenerative diseases [4,5]. Therefore, AD patient exhibit marked decline in cognitive functions and severe behavioral abnormalities such as irritability, aphasia, apraxia, agnosia and restlessness. However, some phytochemicals from natural plant sources like fruits and vegetables may reduce the risk of AD because of their antioxidant properties diminishing oxidative insults [6].

Garlic (*Allium sativum*), a member of the lily family, has long been used as a medicinal food ingredient with physiological potential for a long time. In addition, garlic is one of the most widely grown vegetable crops in Asia including China and is also known for its therapeutic uses and as a flavouring agent since ancient times [7]. Previous report showed that garlic together with vitamins E and C reduced the incidence of precancerous gastric lesions in a large population in China [8]. Garlic is also considered an effective scavenger of free radicals against various diseases caused by ROS. However, many people cannot freely eat fresh raw garlic (FRG) because of its intense taste and smell even though they know that garlic is good for their health. In addition, the consumption of FRG is often associated with several health hazards, such as stomach and digestion problems [7]. Therefore, different formulations of garlic preparation including aged garlic extract (AGE), dehydrated garlic powder, garlic oil and garlic oil macerate was developed [9].

Aged garlic which is recently available on the market in Korea is one of garlic products expected to have strong antioxidant capacity. It has a soft fruity taste with a non-irritating odor. Aged garlic is produced by ageing whole garlic at high temperature (70°C) and high humidity (90% RH) [10]. During ageing process, unstable compounds of fresh garlic including alliin are converted into stable compound including S-allyl cysteine (SAC) and various water soluble compounds with potent antioxidant effect [11]. It was reported that aged garlic contained increased content of total phenolics and stronger *in vitro* antioxidant activity than raw-garlic [10]. Although aged garlic extract has already been demonstrated that an S-allyl-cysteine as an active compound of aged garlic extract has physiological activities including anti-amnesic effects [12], little is known about learning and memory improving effect of resources due to aged garlic extract. Therefore, the present study was undertaken to investigate beneficial effects of aged garlic extract on Aβ-induced cognitive dysfunction in neuron like PC 12 cells and mice.

## Methods

### Materials

2,2'-azino-bis(3-ethylbenzthiazoline-6-sulfonic acid) diammonium salt (ABTS), potassium persulfate, vitamin C, α-tocopherol, catechin, 2-[4-(2-hydroxyethyl)piperazin-1-yl]ethanesulfonic acid (HEPES), sodium bicarbonate, streptomycin, ferrous sulfate (FeSO_4_), hydrogen peroxide (H_2_O_2_), dimethyl sulfoxide (DMSO), penicillin, amyloid β protein (Aβ_25-35_, A4559), 3-[4,5-dimethythiazol-2-yl]-2,5-diphenyl tetrazolium bromide (MTT) assay kit, lactate dehydrogenase (LDH) assay kit were purchased from Sigma Chemical Co (St. Louis, MO, USA). All solvents used were of analytical grade. RPMI 1640 medium and fetal bovine serum was obtained from Gibco BRL (Grand Island, NY, USA).

### Sample preparation

Aged garlic (*Allium sativum*) was supported by Namhae Garlic Research Institute (Namhae, Korea), in December 2010 and was authenticated by Dr. JH Shin (Namhae Garlic Research Institute). Extraction of aged garlic was prepared by pouring 80% ethanol 3.5 L into the bottle containing 1 kg of aged garlic, and the mixture was steeped at room temperature (20°C) for 7 days [13], and filtrated. The extract was then concentrated under reduced pressure at 60°C using vacuum rotary evaporator. Ethanol extract was sequentially partitioned with *n*-hexane, chloroform, ethyl acetate and water. Four solvent fractions were collected and concentrated with vacuum rotary evaporator. The fractions were frozen and lyophilized. Finally, the fractions were placed in a glass bottle and stored at -20°C until used.

### PC12 cell culture

Neuron-like PC12 cell line was derived from a transplantable rat pheochromocytoma. The cells respond reversibly to nerve growth factor (NGF) by induction of the neuronal phenotype. Cells (KCLB 21721, Korea Cell Line Bank, Seoul, Korea) were propagated in RPMI 1640 medium containing 10% fetal bovine serum, 50 units/mL penicillin and 100 μg/mL streptomycin.

### ABTS radical-scavenging activity

ABTS was dissolved in water to make a concentration of 7 mM. ABTS was produced by reacting the ABTS stock solution with 2.45 mM potassium persulfate (final concentration) and allowing the mixture to stand in the dark at room temperature for 12-16 h before use. For the study of samples, the ABTS stock solution was diluted with phosphate-buffered saline 5 mM, pH 7.4 to an absorbance of 0.70 at 734 nm. After the addition of 980 μL of diluted ABTS to 20 μL of sample, the absorbance reading was taken 5 minutes after the initial mixing [14]. This activity is given as percent ABTS scavenging that is calculated as:

$$\begin{array}{l} \%\;\mathrm{ABTS}\;\mathrm{scavenging}\;\mathrm{activity}\\ \;=\left[\left(\mathrm{control}\;\mathrm{absorbance}-\mathrm{sample}\;\mathrm{absorbance}\right)/\right)\\ \;\left(\left(\mathrm{control}\;\mathrm{absorbance}\right)\right]\times 100 \end{array}$$

### Malondialdehyde (MDA) assay using mouse whole brain homogenates

This assay was carried out to the method described by Jeong et al [15]. The brain of young adult male institute of cancer research (ICR) mice were dissected and homogenized in ice-cold Tris–HCl buffer (20 mM, pH 7.4) to produce a 1/10 homogenate. The homogenate was centrifuged at 12,000 × g for 15 minutes at 4°C. 1 mL aliquots of the supernatant were incubated with the test samples in the presence of 10 μM FeSO_4_ and 0.1 mM vitamin C at 37°C for 1 h. The reaction was terminated by addition of 1.0 mL trichloroacetic acid (TCA) (28%, w/v) and 1.5 mL thiobarbituric acid (TBA) (1%, w/v) in succession, and then the solution was heated at 100°C. After 15 minutes, the color of the MDA-TBA complex was measured at 532 nm. (+)-Catechin, a well-known antioxidant, was used as a positive control. The inhibition ratio (%) was calculated as follows:

$$\begin{array}{l} \%\;\mathrm{inhibition}\\ \;=\left[\left(\mathrm{absorbance}\;\mathrm{of}\;\mathrm{control}-\mathrm{absorbance}\;\mathrm{of}\;\mathrm{sample}\right)/\right)\\ \;\left(\mathrm{absorbance}\;\mathrm{of}\;\mathrm{control}\right]\times 100 \end{array}$$

### Measurement of cellular oxidative stress

Levels of cellular oxidative stress were measured by 2',7'-dichlorofluorescein diacetate (DCF-DA). PC12 cells were pretreated with various concentrations of aged garlic for 48 h. Freeze-dried ethyl acetate fraction from aged garlic of various concentrations was dissolved in deionized distilled water. Cells were then treated with or without Aβ_25-35_ for 2 h. At the end of the treatment, cells were incubated in the presence of 50 μM DCF-DA for 50 min. After incubation, 2',7'-dichlorofluorescein (DCF) was quantified using a fluorometer (infinite F200, TECAN, NC, USA) with a 485 nm excitation filter and a 535 nm emission filter. The results were expressed as percent relative to the oxidative stress level of the control cells, which was set to 100% [3].

### Determination of cell viability

MTT reduction assay was determined using the *in vitro* toxicology assay kit. Neuronal PC12 cells were plated at a density of 10^5^ cells/well on 96-well plates in 100 μL of RPMI. Synthetic Aβ_25-35_ was prepared at a concentration of 1 mM in PBS. The cells were pre-incubated with various fractions obtained from aged garlic for 48 h before adding of 80 μM Aβ_25-35_. The cells were treated with or without Aβ for 3 h. MTT reduction was initiated by adding 10 μl MTT stock solution per well. Plates were incubated at 37°C. After 3 h incubation, the reaction was stopped by adding 100 μl of DMSO. The amount of cellular MTT formazan product was determined by measuring absorbance using a microplate reader (680, Bio-Rad, Tokyo, Japan) at a test wavelength of 570 nm and a reference wavelength of 690 nm [16].

Neuronal PC12 cells were precipitated by centrifugation at 250 × *g* for 4 minutes at room temperature, 100 μL of the supernatants was transferred into new wells, and LDH was determined using the *in vitro* toxicology assay kit. Damage of the plasma membrane was evaluated by measuring the amount of the intra-cellular enzyme LDH released into the medium [3].

### Animals

ICR mice (male, 4 weeks old) were obtained from Samtako Co. (Osan, Korea). The mice were housed two per cage in a room maintained with a 12 h light–dark cycle, 55% humidity, and 23-25°C temperature. All animals and experimental procedures were approved by the guidelines established by the 'Institutional Animal Care and Use Committee (IACUC) of Gyeongsang National University (certificate: GNU-120409-M0009), and in accordance with the Ethical Committee of Ministry of Health and Welfare, KOREA. Freeze-dried ethyl acetate fraction from aged garlic extract was mixed in water at concentrations of 5, 10, and 20 mg/kg of body weight. Mice were divided into five groups: (I) control (*n =* 8), (II) Aβ (*n =* 8), (III) Aβ + sample 5 mg/kg (*n =* 8), (IV) Aβ + sample 10 mg/kg (*n =* 8) and (V) Aβ + sample 20 mg/kg (*n =* 8). The mice were allowed free access (*ad libitum*) to water, in which the fraction had been dissolved for 3 weeks. Aβ_25-35_ was administered by intracerebroventricular (ICV) injection to induce memory impairment. Aβ was dissolved in 0.85% (v/v) sodium chloride solution. Each mouse was injected at the bregma with a Hamilton microsyringe (depth, 2.5 mm; injection volume, 5 μL; dose, 410 pmol per mouse) [3].

### Y-maze test

Recording spontaneous alternation behavior in the Y-maze test was used to assess the immediate working memory performance. The Y-maze test was performed 2 days after the ICV Aβ injection. The maze was made of black-painted plastic, and each arm of the maze was 33 cm long, 15 cm high and 10 cm wide, and was positioned at a constant angle. Each mouse was placed at the end of one arm and allowed to move freely through the maze for 8 min. The series of arm entries was recorded visually, and arm entry was considered to have been completed only when the hind paws of the mouse were placed completely in the arm of the maze. Alternation is defined as successive entries into the three arms in an overlapping triplet set. The percentage alternation was calculated as the ratio of actual to possible alternation (defined as the total number of arm entries minus two), multiplied by 100 [3,17].

### Passive avoidance test

The passive avoidance test box was divided into two compartments, one illuminated and one dark, with a grid floor. The mice were allowed to move freely through a circular tunnel between the two compartments. During the training trial (3 days after the ICV Aβ injection), each mouse was placed in the lighted compartment: as soon as it entered the dark compartment, an inescapable electric shock was provided (0.5 mA, 1 sec). In the probe trial (4 days after the ICV Aβ injection), which was given 1 day after the training trial, the mouse was again placed in the lighted compartment, and the time until it re-entered the dark compartment was measured (the step-through latency maximum testing limit was 300 sec) [3].

### Identification of active compounds

Sulfur compounds in fractions obtained from aged garlic were measured at 195 nm by a photo diode array detector (Ultimate 3000 series, Dionex, CA, USA). Separation was achieved with a Shiseido C_18_ column (250 mm × 4.6 mm id, 5 μm, Shiseido Co., Tokyo, Japan). The mobile phase consisted of water (A) and acetonitrile (B). The linear gradient as reverse phase condition was 0-60% (B) in 20 min, at a flow rate of 0.5 mL/min and the injection volume was 20 μL. Main sulfur compounds in aged extract were identified by comparison of their retention time (RT) values and UV spectra of known standards.

### Statistical analysis

All data were expressed as mean ± SD. Each experimental set was compared with one-way analysis of variance (ANOVA) and Duncan’s multiple-range test (*P* < 0.05) using SAS program (SAS Institute, Cary, NC, USA).

## Results

### ABTS radical scavenging activities and inhibition of lipid peroxidation

**Figure 1:**
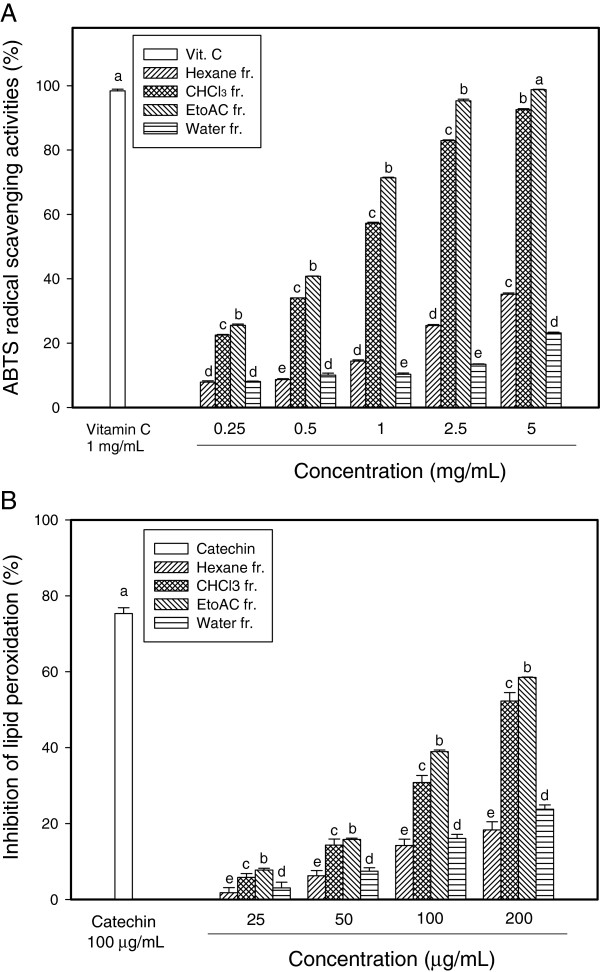
**Antioxidant activities.** ABTS radical scavenging activities **(A)** and Inhibition effect of ferric ion and vitaminC-induced lipid peroxidation on mouse brain homogenates **(B)** of various fractions from aged garlic extracts**.** Each value represented the means ± SD of triplicates. Different superscripts indicate significant difference among groups at *p <* 0.05.

The reduction capability of ABTS radical was determined by the decrease in its absorbance at 734 nm, which is induced by antioxidants. Positive ABTS test suggests that aged garlic extracts were free radical scavengers. The extracts exhibited ABTS radical scavenging activities to different extents a concentration-dependent manner. The ethyl acetate fraction exhibited the highest radical scavenging activities when they reacted with the ABTS radical. Although the activity levels of *n*-hexane, chloroform and water were lower than that of ascorbic acid. ABTS radical scavenging effect of the ethyl acetate extracts at 5 mg/mL, which was similar to that of 1 mg/mL vitamin C (*p* < 0.05) (Figure 1A). In contrast, the *n*-hexane and water extracts only showed low activities; approximately 3 times lower than the ethyl acetate fraction.

There has been an increasing interest in lipid peroxidation because formation of cytotoxic products such as MDA and 4-hydroxynonenal can influence cellular apoptosis, and several human diseases [18]. Therefore, in this assay, antioxidant activities of aged garlic extracts on vitamin C-induced lipid peroxidation on mouse whole brain homogenates were also confirmed. Figure 1B revealed that the ethyl acetate fraction have excellent activities in suppressing lipid peroxidation on mouse whole brain homogenates. Although the ethyl acetate fraction presented lower than catechin as a positive control at all concentration, it was also noteworthy that more than 50% of inhibitory activity of lipid peroxidation was observed at the concentration of 200 μg/mL.

### Measurement of cellular oxidative stress

**Figure 2:**
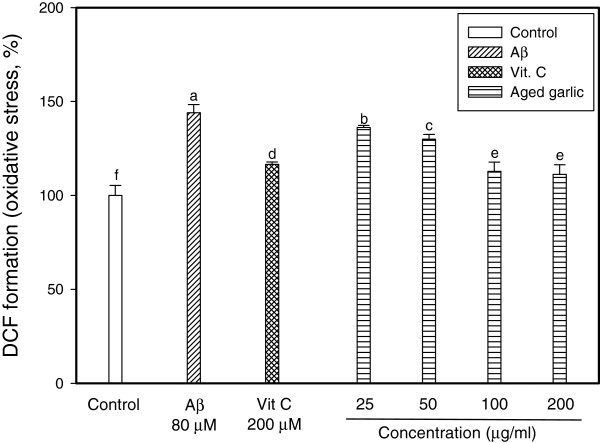
**Effect of ethyl acetate fraction from aged garlic extract on ROS production determined in the presence and absence of Aβ in PC12 cell.** PC12 cells were pretreated for 48 h with various concentrations. After 48 h, the cells were treated with 80 μM Aβ for 2 h. The increase of DCF-DA fluorescence was measured by fluorescence microplate reader. Each value represented the means ± SD of triplicates. Different superscripts indicate significant difference among groups at *p <* 0.05.

The ethyl acetate fraction obtained from aged garlic extract showed prevention of cellular ROS accumulation. Exposure of PC12 cells to Aβ for two hours resulted in a 144% increase of oxidative stress levels compared to the control (Figure 2). Pretreatment of PC12 cells by ethyl acetate fraction from aged garlic extract significantly prevented them from intracellular ROS accumulation in comparison to the PC12 cells treated only with Aβ. PC12 cells at the level of 200 μM vitamin C as a positive control had significantly lower oxidative stress than PC12 cells with treatments of Aβ only (Figure 2). In particular, the groups of treatment with ≥ 100 μg/mL showed similar inhibition of oxidative stress to vitamin C (*p* < 0.05). In this respect, this result suggests that the aged garlic with antioxidant activity may play an important role to reduce the cellular oxidative stress, which is an important risk factor for neurodegenerative diseases such as AD.

### PC12 cell protection against Aβ-induced neurotoxicity

**Figure 3:**
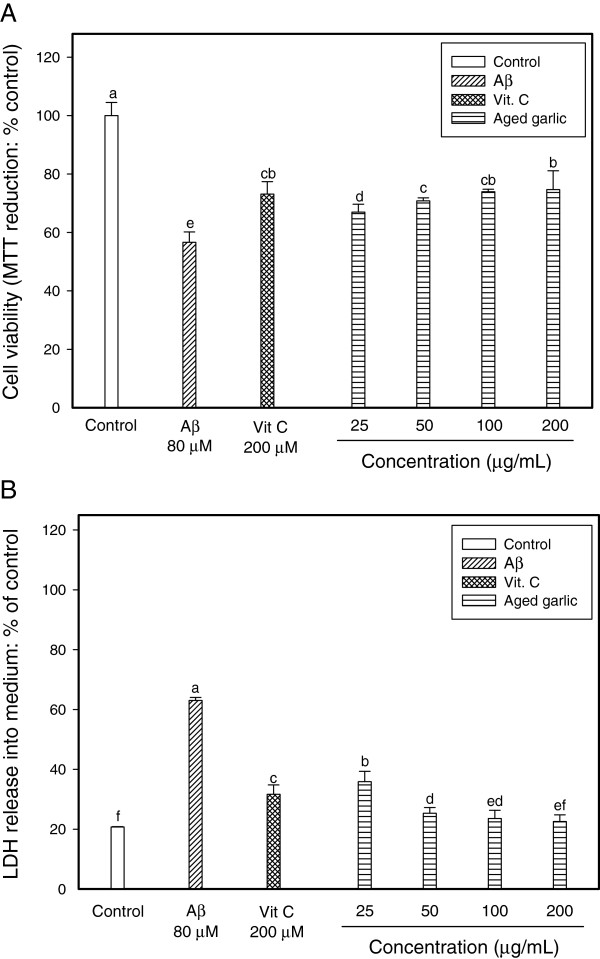
**Neuroprotective effect of ethyl acetate fraction from aged garlic extract on Aβ-induced cytotoxicity in PC12 cells. (A)** Protective effect of the ethyl acetate fraction on Aβ-induced cytotoxicity in PC12 cell system. PC12 cells were pretreated for 24 h with various concentrations. After 24 h, the cells were treated with 80 μM Aβ for 3 h. **(B)** Inhibitory effect of the ethyl acetate fraction on Aβ-induced membrane damage in PC12 cells. PC12 cells were pretreated for 48 h with various concentrations. After 48 h, the cells were treated with 80 μM Aβ for 3 h. Each value represented the means ± SD of triplicates. Significant difference (*p <* 0.05 versus vitamin C) was observed on the Aβ-induced cell death.

The MTT assay relies primarily on the mitochondrial metabolic capacity of viable cells and reflects the intracellular redox state. Cultured PC12 cells were treated with Aβ_25--35_ for 3 h, and the Aβ-induced neurotoxic effect was examined by the MTT reduction assay. Aβ treatment caused a decrease in cell viability (80 μM; about 56%). Pretreatment cell with ethyl acetate fraction from aged garlic extracts inhibited Aβ-induced cytotoxicity (Figure 3A). PC12 cell protective effect of the ethyl acetate fraction at all concentration on oxidative injury was similar to that of 200 μM vitamin C (*p* < 0.05). This study demonstrated that PC12 cell cytotoxicity through Aβ_25-35_-induced neurotoxicity was suppressed by pretreatment with ethyl acetate fraction obtained from aged garlic.

To investigate the probability of Aβ-induced membrane damage, we have assessed the protective effect of the ethyl acetate fraction using the LDH assay, measuring the activity of this stable enzyme released into the medium from apoptotic PC12 cells. LDH assay provided an estimate of the percentage of surviving PC12 cells. The ethyl acetate fraction protected the integrity of the cellular membrane at all the concentrations tested (Figure 3B). With Aβ_25--35_ treatment for 3 h, the amounts of LDH release of PC12 cells increased up to 42% compared to that of the control without treatment. Our results indicate that ethyl acetate fraction from aged garlic extract protects the PC12 cell membrane against Aβ-induced neurotoxicity.

### Anti-amnesic activities against ICV Aβ injection

**Figure 4:**
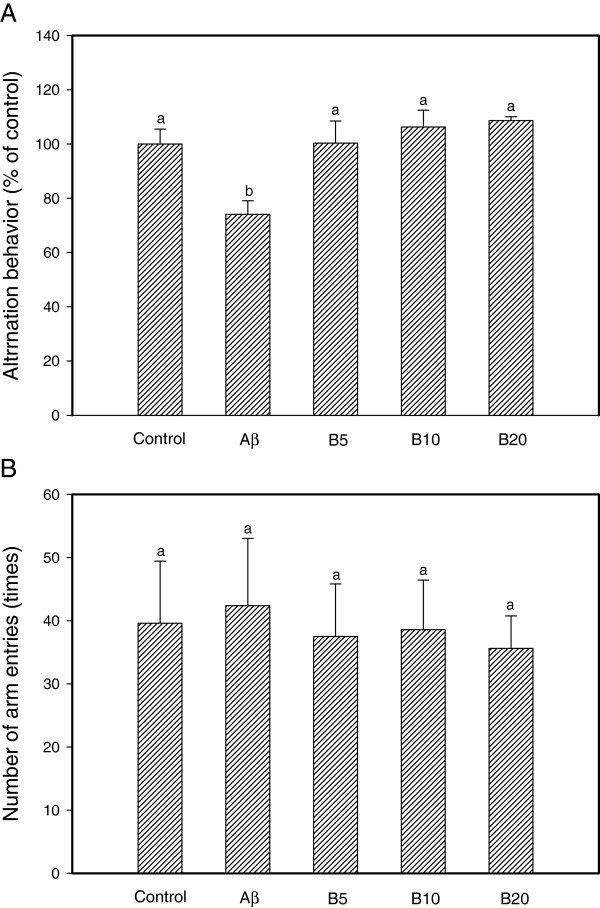
**Effect of ethyl acetate fraction from aged garlic extract on spontaneous alternation behavior.** Control group was injected with saline (0.85%). Aβ_25-35_ was injected with 410 pmol of Aβ_25-35_ per mouse. Sample groups were injected Aβ_25-35_ followed by feeding with the ethyl acetate fraction (B5-B20: 5, 10 and 20 mg/kg per day, respectively). The sample was dissolved in water. The spontaneous alternation behavior **(A)** and number of arm entries **(B)** of Y-maze were measured over 8 min. Values indicate the mean ± SD (n = 8). *p <* 0.05 versus control group, *p <* 0.05 versus Aβ_25-35_ group. Values with the same letter are not significantly different.

The effect of dietary administration of ethyl acetate fraction from aged garlic extract on anti-amnesic effect perception was examined using an AD animal model based on an ICV Aβ injection. Memory and learning abilities were evaluated in a Y-maze test and a passive avoidance task. In Figure 4A, the Aβ_25--35_ injected mice exhibited a significantly impaired spatial working memory (25.93% decrease in alternation behavior) compared with that of control group (100%). The groups which were pretreated with the sample increased spontaneous alternation behavior after Aβ injection (the ethyl acetate fraction 5 mg/kg: B5, 100.30%; 10 mg/kg: B10, 106.33%; 20 mg/kg: B20, 108.60%). In contrast, the number of arm entries did not change among all the experimental group, which demonstrated that general locomotor activity was not affected by Aβ_25--35_ (Figure 4B).

**Figure 5:**
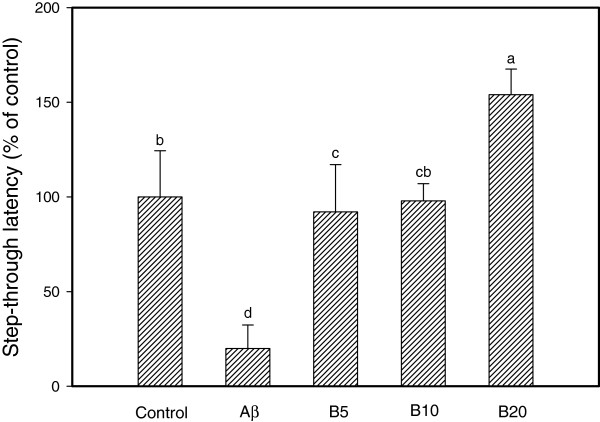
**Protective effects of ethyl acetate fraction from aged garlic extract against Aβ-induced memory impairment in mice.** Control group was injected with saline (0.85%). Aβ_25-35_ was injected with 410 pmol of Aβ_25-35_ per mouse. For further explanation see the legend to Figure 4. Values indicate the mean ± SD (n = 8). *p <* 0.05 versus control group, *p <* 0.05 versus Aβ_25-35_ group. Values with the same letter are not significantly different.

As shown in Figure 5, mice treated with ethyl acetate fraction from aged garlic extract exhibited attenuated Aβ-induced impairment in a dose-dependent manner. The passive avoidance test was carried out 4 days after Aβ_25--35_ injections. The Aβ injected mice displayed a significant reduction (an 81.07% decrease) in step-through latency compared with the control group. Ethyl acetate fraction from aged garlic extract attenuated the Aβ-induced impairment of mice in the passive avoidance test. Therefore, these results indicate that ethyl acetate fraction from aged garlic extract displayed a significant anti-amnestic effect in the AD mouse model.

### Analysis of sulfur compounds by high performance liquid chromatography (HPLC)

**Figure 6:**
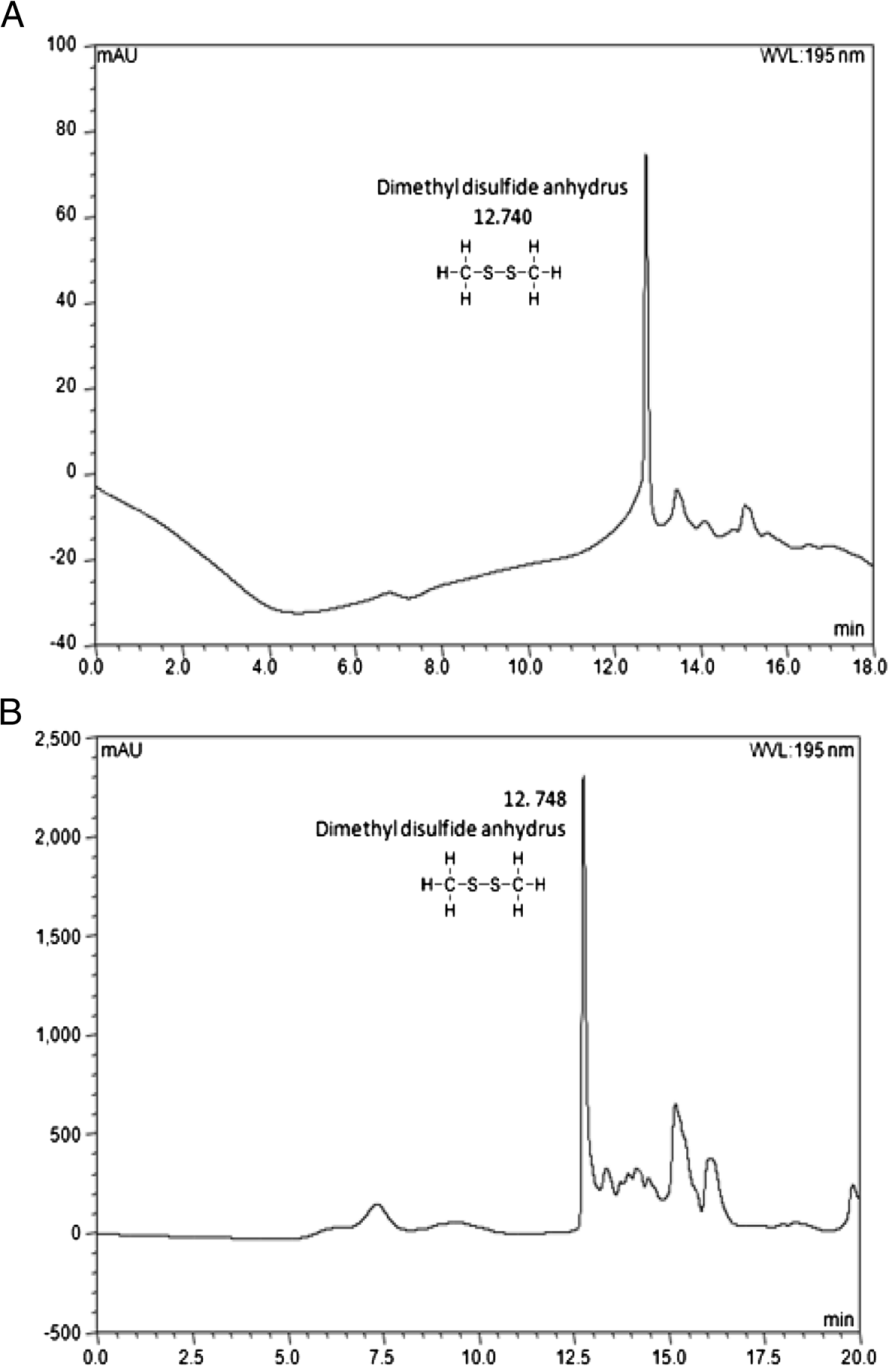
**HPLC chromatogram of ethyl acetate fraction from aged garlic.** HPLC chromatogram of standards **(A)** and ethyl acetate fraction **(B)** from aged garlic at 195 nm.

Since the ethyl acetate fraction exhibited the strongest antioxidant activity, PC12 cell protective activity and *in vivo* ameliorating effect, it was subjected to further analysis by HPLC to find physiological main compounds. By comparing the retention time and UV spectra of these compounds with those of standard, a dimethyl disulfide anhydrus as main thiosulfate was identified (Figure 6). Consequently, depending on the versatile extraction conditions, the main ingredient of garlic extract will be variously presented sulphide compounds such as a dimethyl disulfide which was identified in the ethyl acetate fraction of aged garlic extract.

## Discussion

Antioxidants can be referred to as reductants, which are inactivating oxidants. They are involved in redox reactions in which on reaction species (oxidant) is reduced at the expense of the oxidation of the antioxidant (reductant) [19]. Oxidative stress caused by increased accumulation of ROS has been implicated in neurodegenerative diseases such as AD [20]. Oxidative stress in AD may result from aging, energy deficiency, inflammation or excessive production of Aβ. Amyloid β protein can induce cell death through a mechanism involving hydrogen peroxide [20,21]. Neurotoxic properties are characteristic not only of the whole Aβ peptide Aβ_1-40(42)_, but also of a number of fragments, the most neurotoxic of which is fragment Aβ_25-35_. Furthermore, Aβ_25–35_ is more rapidly toxic and causes more oxidative damage than the parent peptide Aβ_1–42_. The neurotoxic activity of Aβ has been attributed to amino acids present in positions 25-35 of the full-length beta-amyloid [22-24]. Intracellular oxidative stress levels were evaluated by measuring fluorescent DCF formation in PC12 cells. The foregoing experiments, intracellular oxidative stress resulting from Aβ treatment was significantly lower when PC12 cells were treated with the ethyl acetate fraction from aged garlic extract compared with cells treated only with Aβ. These results demonstrate that the ethyl acetate fraction from aged garlic extract protected PC12 cells from Aβ_25-35_-induced ROS generation *in vitro*.

Deposits of Aβ and neurofibrillary tangles are the two pathological hallmarks of AD. There is recent evidence that Aβ aggregates can impair function, morphology and subsequently the viability of neuronal cells [21]. Amyloid β protein, in the form of insoluble fibril deposits, is the important constituent of senile plaques in AD patients, and it has been suggested to be the cause of the neurodegeneration that occurs in AD brains [2]. To evaluate Aβ neurotoxicity properly, it is important to employ an appropriate method for quantitating cell viability. Aβ_25-35_ has neurotoxicity and produces free radical adducts in aqueous solutions and sensitizes neurons to injury resulting from oxidative stress-induced neurotoxicity induced by glutamate or free radicals [25]. The assay with MTT, a yellow water-soluble tetrazolium salt, is a simple colorimetric assay to measure cell cytotoxicity. This assay has been shown repeatedly to be a very sensitive indicator of the cell death induced by Aβ [2]. Our data showed that Aβ_25-35_ caused a decrease in cell viability, but pretreating ethyl acetate fraction from aged garlic extract inhibited Aβ-induced neurotoxicity in a dose-dependent manner. Oh et al. [22] also treated PC12 cells with Aβ_25-35_, and measured cell viability by an MTT reduction assay. The viability of PC12 cells after treatment with Aβ_25-35_ (20–80 μM) for 48 h decreased in a dose-dependent manner to 82.0% (20 μM), 53.0% (40 μM), 48.8% (60 μM), and 40.4% (80 μM). Therefore, these results demonstrate that PC12 cell protection by ethyl acetate fraction from aged garlic extract is in part due to the mitochondrial protection mechanism.

Lipid peroxidation is increased in neurodegenerative diseases such as AD. Polyunsaturated fatty acid levels, especially arachidonic acid and docosahexaenoic acid, are high in neuronal cells of brain. They are more vulnerable to attack by ROS, peroxidation of which can lead to changes in membrane integrity and fluidity [16]. Because the neuronal plasma membrane is sensitive to oxidative stress, the cell membrane protective effect of ethyl acetate fraction from aged garlic extracts on Aβ-induced neurotoxicity was investigated by the LDH release assay, measuring the activity of this stable enzyme released into the medium from apoptotic PC 12 cells. Our results indicate that ethyl acetate fraction from aged garlic extract protects the PC12 cell membrane against Aβ-induced neurotoxicity.

The Aβ_25–35_-induced disruption in hippocampal network activity correlates with a reduction in spontaneous neuronal activity and synaptic transmission, as well as with an inhibition in the sub-threshold oscillations produced by pyramidal neurons *in vitro*[23]. The effect of dietary administration of ethyl acetate fraction from aged garlic extract on behavioral abilities was examined using and AD animal model based on an intracerebroventricular Aβ injection. Memory and learning abilities were evaluated in a Y-maze test and a passive avoidance task. The mouse model was used to measure Aβ-induced memory impairment. This suggests that in mice, exposure to the Aβ fragment cause impairments in the learning and memory systems. It has also been reported that antioxidants can protect against Aβ-peptide-induced toxicity [3]. In this study, we have shown that in mice, the Aβ_25-35_-induced cognitive deficits exerted via various cytotoxicities including oxidative stress and disruption of hippocampal network activity are suppressed by pretreatment with aged garlic.

The major volatile compounds of garlic were sulfur containing compounds. Okada [26] studied structure antioxidant activity relationship for thiosulfinates and suggested that the combination of the allyl group (–CH_2_CH = CH_2_) and the –S(O)S– group is necessary for the antioxidant action of thiosulfinates in the garlic extract. Major volatiles of raw and heated garlic were dimethyl disulfide, 2-propen-1-ol, allyl methyl disulfide, dimethyl trisulfide, diallyl disulfide, allyl methyl trisulfide, and diallyl trisulfide. However, a dimethyl disulfide (DMDS), which was not observed in raw garlic samples, was just observed in heated samples (autoclaved garlic clove and autoclaved-crushed), indicating thermal reaction may cause the formation of this compound [27]. Storing sliced raw garlic in 15%–20% ethanol for 20 months produces AGE. This whole process is supposed to cause a considerable loss of allicin and to increase the activity of certain newer compounds, such as SAC, S-allylmercaptocysteine, allixin and selenium which are stable, highly bioavailable and significantly antioxidant [28]. Our study demonstrated that PC12 cell cytotoxicity through Aβ_25-35_-induced neurotoxicity was suppressed by pretreatment with ethyl acetate fraction obtained from ethanolic extract of aged garlic. In our research, we showed that ethyl acetate fraction form ethanolic garlic extract had *in vitro* antioxidant activities, PC12 cell protections, and *in vivo* anti-amnesic effects. Ito Y et al. [29] also demonstrated that SAC also protected cultured hippocampal neurons against Aβ-induced neuronal death. In this study, we have shown that aged garlic extract containing various compounds with DMDS is considered that may be helpful to overcome Aβ-induced cognitive impairment and cytotoxicity.

DMDS was identified as one with greater concentration toward the mix of volatiles. The abundance (%) of DMDS was about 30% in volatile compounds from S-methyl cysteine sulfoxide. However contents of DMDS may be changed, depending on samples (fresh garlic, aged garlic, and aged garlic oil etc.) [11,30]. Since the cumulative concentration of organosulfides which was consumed in aged garlic extracts is high, it is likely that the combined concentrations of ingested organosulfides may reach levels high enough to bring about a cellular response and cognitive improvement in mice.

## Conclusions

Anti-amnesic effects of ethyl acetate fraction from aged garlic extract on learning and memory deficits were evaluated with *in vitro/vivo* animal models. We discovered from our present work that among fractions of the extract, the ethyl acetate fraction has the highest levels of in *vitro* antioxidant activities and neuroprotective effect against Aβ-induced cytotoxicity in neuron-like PC12 cells. Moreover, biochemical experiments using whole brain tissues clearly showed lowered oxidative stress levels, and ethyl acetate fraction from aged garlic extract attenuated both memory and cognitive ability against Aβ-induced deficits. Therefore, the physiological activities of ethyl acetate fraction from aged garlic extracts may be attributed to sulfide compounds which include dimethyl disulfide anhydrus.

## Abbreviations

ABTS: 2,2'-azino-bis(3-ethylbenzthiazoline-6-sulfonic acid) diammonium salt; MDA: Malondialdehyde; ROS: Reactive oxygen species; DCF-DA: 2',7'-dichlorofluorescein diacetate; MTT: 3-[4,5-dimethythiazol-2-yl]-2,5-diphenyl tetrazolium bromide; LDH: Lactate dehydtrogenase; ICR: Institute of cancer rese; Aβ: Amyloid beta protein; AD: Alzheimer's disease; AGE: Aged garlic extract; FRG: Fresh raw garlic; DMSO: Dimethyl sulfoxide; NGF: Nerve growth factor; TCA: Trichloroacetic acid; TBA: Thiobarbituric acid; IACUC: Institutional animal care and use committee; HPLC: High performance liquid chromatography; RT: Retention time; ICV: Intracerebroventricular; SAC: S-allyl cysteine; DMDS: Dimethyl disulfide.

## Competing interests

The authors declare that they have no competing interests.

## Authors’ contributions

HJH participated in the design of the study. JHJ, HRJ, YNJ and HJK conducted the experiments, analyzed the data and drafted the manuscript. JHS helped conduct the experiments. All authors read and approved the final version of the manuscript.

## Pre-publication history

The pre-publication history for this paper can be accessed here:

http://www.biomedcentral.com/1472-6882/13/268/prepub
